# Point-of-care multiplex PCR promises short turnaround times for microbial testing in hospital-acquired pneumonia – an observational pilot study in critical ill patients

**DOI:** 10.1186/s12941-015-0091-3

**Published:** 2015-06-13

**Authors:** Nils Kunze, Onnen Moerer, Nicolas Steinmetz, Marco H. Schulze, Michael Quintel, Thorsten Perl

**Affiliations:** Department of Anaesthesiology, University Medical Centre, University of Göttingen, Robert-Koch-Straße 40, Göttingen, 37075 Germany; Institute for Medical Microbiology, University Medical Centre, University of Göttingen, Kreuzbergring 57, Göttingen, 37075 Germany

**Keywords:** Pneumonia, Point-of-care systems, Microbiological techniques, Multiplex polymerase chain reaction, Molecular diagnostic techniques, Drug resistance

## Abstract

**Background:**

The early beginning of an adequate antibiotic therapy is crucial in hospital-acquired pneumonia (HAP), but depends on the results of conventional microbiological diagnostics (cMD). It was the aim of this study to evaluate the performance and turnaround times of a new point-of-care multiplex polymerase chain reaction (mPCR) system for rapid identification of pathogens and antibiotic resistance markers. We assessed the applicability of the system under real-life conditions in critical ill patients with HAP.

**Methods:**

We enrolled forty critical ill patients with clinical signs for HAP into an observational study. Two samples of respiratory secretions were collected during one course of aspiration and cMD and mPCR testing (Unyvero, Curetis AG, Holzgerlingen, Germany) were performed immediately. The mPCR device was operated as a point-of-care system at the intensive care unit. We compared turnaround times, results of pathogen identification and results of antibiotic resistance testing of both methods.

**Results:**

Mean turnaround times (min-max) were 6.5 h (4.7–18.3 h) for multiplex PCR and 71 h (37.2–217.8 h) for conventional microbiology (final cMD results, incomplete results neglected). 60 % (*n* = 24) of the mPCR tests were completely valid. Complete test failure occurred in 10 % (*n* = 4) and partial test failure occurred in 30 % (*n* = 12). We found concordant results in 45 % (*n* = 18) and non-concordant results in 45 % (*n* = 18) of all patients. 55 % (*n* = 16) of the results were concordant in patients with a clinical pulmonary infection score (CPIS) > 5 (*n* = 29). Concordant results included three cases of multidrug resistant bacteria. MPCR frequently detected antibiotic resistance markers that were not found by cMD.

**Conclusions:**

Unyvero allowed point-of-care microbial testing with short turnaround times. The performance of the system was poor. However, an improved system with a more reliable performance and an extended microbial panel could be a useful addition to cMD in intensive care medicine.

**Trial registration:**

ClinicalTrials.gov NCT01858974 (registered 16 May 2013)

## Background

Hospital-acquired pneumonia (HAP) is the second most common nosocomial infection in the intensive care unit (ICU) and accounts for about 25 % of all infections [1]. Patients receiving mechanical ventilation are at particular risk for developing lower respiratory tract infections. Mortality, morbidity and therapy costs are increased in patients suffering from HAP [1]. Early and adequate antibiotic therapy has major impact on the outcome of these patients [2].

The rapid start of an adequate antimicrobial therapy still is foiled by a lack of fast and reliable methods for the identification of the etiologic pathogen. Conventional microbiological diagnostic methods, such as culturing of lower respiratory tract secretions, enable reliable identification of pathogens and their antibiotic resistances. However, with turnaround times between 48 and 72 h these methods are slow and intensive care practitioners are forced to start broad-spectrum antibiotic therapy based on the patient’s risk profile and knowledge of the local patterns of microbes and antibiotic resistance [3]. Besides the risk for an ineffective therapy, such treatment regimen can contribute to the induction of drug-resistances.

A fast and reliable method for the identification of microbes could help to reduce the time to initiate an optimal antibiotic therapy, improve patient’s outcome and lower the risk for the induction of drug-resistances. Genomic, proteomic and metabolomic methods have been proposed for that purpose, but are currently not routinely used [4–6].

With the Unyvero multiplex PCR device (Curetis AG, Holzgerlingen, Germany) a new point-of-care application for the identification of pathogens and their drug resistances directly from respiratory secretions recently was made available.

It was the purpose of this study to evaluate the point-of-care performance of Unyvero in the setting of HAP in the ICU. Therefore we collected specimen of respiratory secretions from ICU patients with clinical diagnosed HAP and compared the results and turnaround times with the performance of conventional microbiological diagnostics (cMD).

## Methods

This observational study was approved by the ethical committee of the Faculty of Medicine of the University of Göttingen (1/7/06, amendment 3) and informed consent was obtained from patients after recovery from acute illness. According to the approval of the ethical committee we did not exclude patients who did not recover and died during their ICU stay. The study was registered at ClinicalTrial.gov (www.clinicaltrials.gov; NCT01858974).

Between April 2013 and January 2014, adult (>18 years) patients from two ICUs of the University Medical Centre of Göttingen were prospectively enrolled.

Inclusion criteria were: Onset of clinical symptoms of pneumonia > 48 h after hospital-admission and sampling of respiratory secretion for microbiological diagnostics under the suspect of pneumonia. The adapted clinical pulmonary infection score (CPIS) was calculated for each patient [7].

Exclusion criteria were: Enrollment for an interventional clinical trial, and suspect for an infection with a pathogen of risk class ≥ 3 according to German law (e.g. *Mycobacterium tuberculosis*).

After study inclusion, two samples of respiratory secretion were collected during one course of direct endotracheal aspiration (ventilated patients) or nasopharyngeal tracheal aspiration (non-ventilated patients). One specimen was sent to the Institute for Medical Microbiology for cMD. Turnaround times for cMD were calculated for the complete final results (including antibiotic resistance testing). We neglected early non-written and/or incomplete cMD results, as we were not able to raise reliable turnaround times for those.

The second specimen was used for Unyvero (Curetis AG, Holzgerlingen, Germany) mPCR testing at the ICU and processed according to the manufacturer’s manual: 180 μl of the aspirate were loaded into a sample tube for pathogen lysis. After 30 min in the Unyvero L4 Lysator the sample tube and the so called *master mix tube* (containing the necessary reactants) were loaded into the self-containing cartridge (Unyvero P50 Pneumonia Cartridge, Curetis AG, Holzgerlingen, Germany) and transferred into the Unyvero A50 Analyzer for further processing, including DNA amplification and detection of the amplified sequences. Detailed information on the working principle of the system can be found on the manufacturer’s website (www.curetis.com). The mPCR system was operated at the ICU. The intensive care physician on duty managed the samples and performed the mPCR tests. For both methods, mPCR and cMD, turnaround times were defined as the time intervals between sampling and the availability of the final and validated results.

Table 1 shows the pathogens (16 bacteria, 1 fungus) and Table 2 shows the 18 resistance markers detected by Unyvero. The ICU physician on duty performed mPCR testing during everyday routine. All personnel performing mPCR tests for the study was instructed in the use of the mPCR device.

**Table 1:** Pathogens detected by the mPCR device (according to the manufacturer)

Gram-positive	Gram-negative	Fungal pathogens
*Streptoccocus pneumoniae*	*Pseudomonas aeruginosa*	*Pneumocystis jirovecii*
*Staphylococcus aureus*	*Acinetobacter baumanii*	
	*Legionella pneumophilia*	
	*Moraxella catarrhalis*	
	*Stenotrophomonas maltophilia*	
	*Enterobacter* species	
	*Escherichia coli*	
	*Klebsiella pneumoniae*	
	*Klebsiella oxytoca*	
	*Proteus* species	
	*Serratia marcescens*	
	*Morganella morganii*	
	*Haemophilus influenzae*	
	*Chlamydophila pneumoniae*

**Table 2:** Resistance markers detected by the mPCR device (according to the manufacturer)

Resistance marker	Resistance against	Relevant pathogen group^a^
mecA	Oxacillin, Methicillin	*Staphylococcus* species
msrA	Macrolides	*Staphylococcus* species
mefA/E	Macrolides	*Streptococcus* species
ermA	Macrolides / Lincosamides	*Staphylococcus* species
ermB	Macrolides / Lincosamides	*Streptococcus* species
ermC	Macrolide / Lincosamides	*Staphylococcus* species
tem	Penicillins, 3rd Gen. Cephalosporins	Enterobacteriaceae, Non-fermenting bacteria, *Haemophilus influenzae*
shv	Penicillins, 3rd Gen. Cephalosporin	Enterobacteriaceae, Non-fermenting bacteria
ctx-M	Penicillins, 3rd Gen. Cephalosporins	Enterobacteriaceae, Non-fermenting bacteria
dha	3rd Gen. Cephalosporins	Enterobacteriaceae
ebc	3rd Gen. Cephalosporins	Enterobacteriaceae
oxa51 like	Carbapenems	*Acinetobacter baumanii*
kpc	Carbapenems	Enterobacteriaceae, Non-fermenting bacteria
int1	Multidrug resistance	Enterobacteriaceae, Non-fermenting bacteria
sul1	Multidrug resistance, Sulfonamides	Enterobacteriaceae, Non-fermenting bacteria
gyrA83	Fluoroquinolones	*Escherichia coli*, *Pseudomonas aeruginosa*
gyrA87	Fluoroquinolones	*Escherichia coli*, *Pseudomonas aeruginosa*
parC	Fluoroquinolones	*Pseudomonas aeruginosa*

^a^ pathogen/group of pathogens in which the resistance marker gene is found (according to the manufacturer)

Descriptive statistical analyses were performed using statistical software (Statistica 10, StatSoft Inc., Tulsa, USA).

## Results

Overall, 62 patients were eligible for the study, of whom 22 were excluded due to missing consent, inaccurate sample collection or missing data. The remaining 40 patients were enrolled and analyzed according to the study protocol. Table 3 shows the characteristics of the study patients.

**Table 3:** Characteristics of the study group

	Patients (*n* = 40)		
Mean age (range)		72 (20–86)	
Female		27.5 % [11]	
Ventilated		65 % [26]	
Death on ICU		35 % [14]	
Mean CPIS^a^ (range)		7 (2–10)	
CPIS Temperature	0 = 47.5 % [19]	1 = 20 % [8]	2 = 32.5 % [13]
CPIS Leucocytosis	0 = 30 % [12]	1 = 70 % [28]	2 = 20 % [8]
CPIS Tracheal secretion	0 = 0 % [0]	1 = 30 % [12]	2 = 70 % [28]
CPIS Chest X-Ray	0 = 7.5 % [3]	1 = 40 % [16]	2 = 52.5 % [21]
CPIS PaO_2_/FiO_2_-Ratio	0 = 17.5 % [7]	-	2 = 82.5 % [33]
CPIS > 5 points		72.5 % [29]	

^a^ CPIS at the time of study inclusion, before results of cultivation were available

[ ] Number of patients

The mean turnaround time for cMD was 71 h (min-max: 37.2–217.8 h). The mean turnaround time for mPCR was 6.5 h (min-max: 4.7–18.3 h).

mPCR provided valid results for one or more areas of the test panel in 36 patients (90 %). Complete test failure occurred in four patients (10 %). Apart from complete failure, one or more area of the test panel failed in twelve patients (30 %). We considered these to be partial test failures. CMD provided valid results in all 40 patients (100 %).

**Fig. 1:**
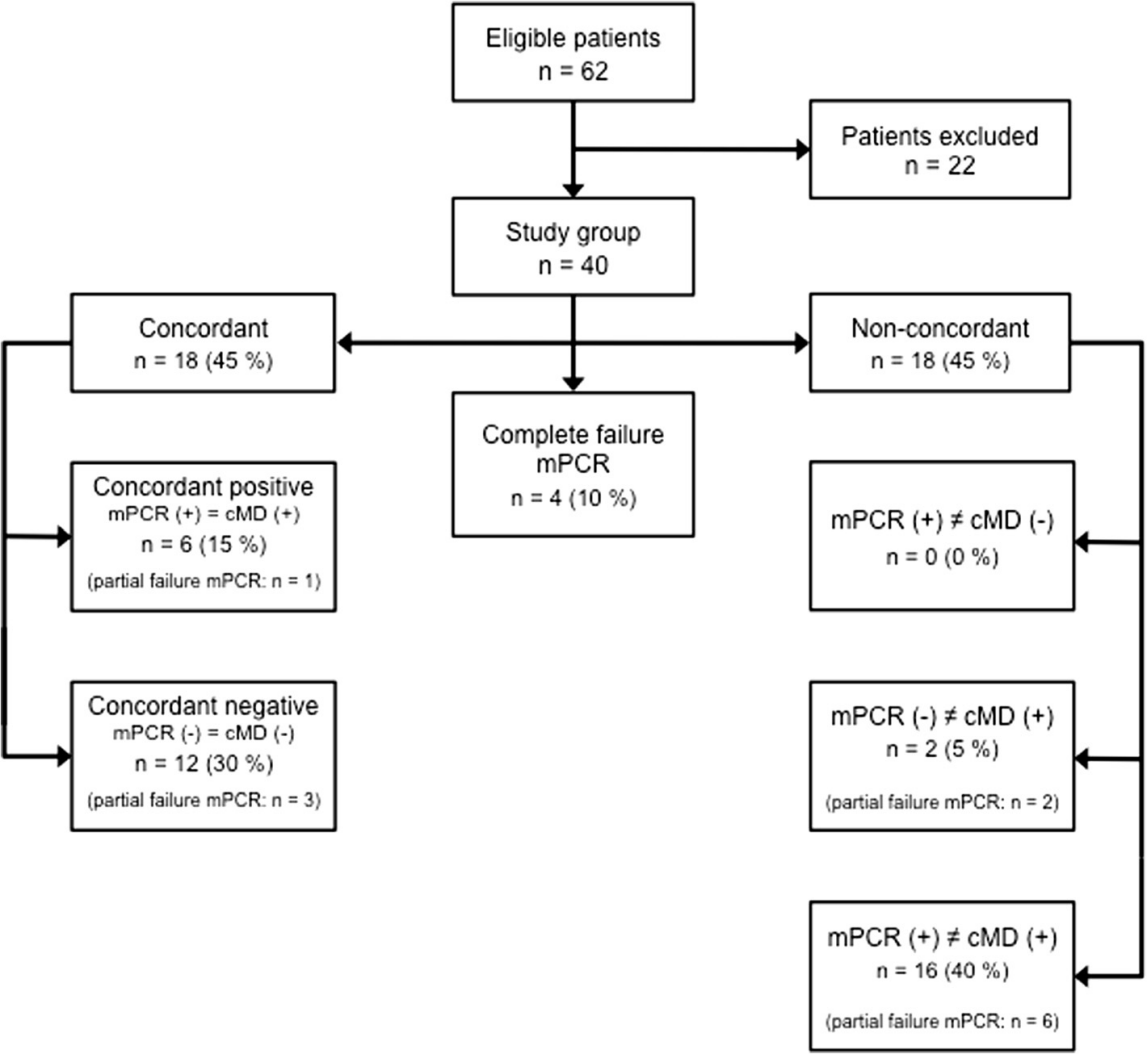
Results of all study patients

In 18 patients (45 %) the results of mPCR were concordant to the results of cMD. Out of these cases, six patients were concordant positive (15 %) and twelve were concordant negative (30 %). Non-concordant results were found in 18 patients (45 %). In two patients (5 %) cMD alone detected a pathogen. In no case only mPCR detected a pathogen while cMD did not detect one. A mismatch between both methods was found in 16 patients (40 %). Figure 1 shows the main results of the study and the distribution of the partly failed mPCR analyses to each group. Table 4 shows the detailed results for all 24 cases with at least one positive result in mPCR or cMD.

**Table 4:** Detailed results for all cases with at least one positive result in mPCR or cMD

Case no.	Results mPCR pathogens	Resistances	Results cMD pathogens	Resistances
*Concordant cases [mPCR (+) = cMD (+)]:*				
1^a^	*Escherichia coli*	ermB, tem^b^, ctx-M^b^, sul1^b^, gyrA83^b^, gyrA87^b^	*Escherichia coli* (3-MRGN)	SAM, TZP, CXM, CTX, CAZ, GM, CIP, MXF, SXT,
2	*Escherichia coli*	mecA, ermC, mefA, ermB, gyrA83^b^, gyrA87^b^	*Escherichia coli*	none
3	*Staphylococcus aureus*	ermC^b^, mefA, sul1	*Staphylococcus aureus*	E, CC
4	*Staphylococcus aureus*	ermB, ermC^b^	*Staphylococcus aureus* (MRSA)	P, OX, SAM, CXM, MXF
5	*Enterobacter species*	ebc^b^	*Enterobacter cloacae*	SAM
6	*Staphylococcus aureus*	mecA^b^, ermB	*Staphylococcus aureus* (MRSA)	P, OX, SAM, CXM, MXF
*Non-concordant cases [mPCR (−) ≠ cMD(+)]:*				
7^a^	none	none	*Enterobacter aerogenes*	none
8^a^	none	int1	*Haemophilus influenzae, Citrobacter koseri*	none, none
*Non-concordant cases [mPCR (+) ≠ cMD(+)]:*				
9^a^	*Moraxella catarrhalis*	ermB	*Moraxella catarrhalis, Klebsiella pneumoniae*	none, AM
10	*Moraxella catarrhalis, Klebsiella pneumoniae, Acinetobacter baumanii*	mecA, ermC, mefA	*Klebsiella pneumoniae, Hafnia alvei*	AM, SAM, TZP
11	*Streptococcus pneumonia, Chlamydia pneumoniae*	mefA^b^, ermB^b^, tem	*Enterobacter amnigenus, Escherichia coli,*	AM, CXM, none
12	*Haemophilus influenzae, Staphylococcus aureus*	mecA^b^, ermB, int1	*Staphylococcus aureus*	P
13	*Streptococcus pneumoniae*	ermC, mefA^b^, ermB^b^, ctx-M, kpc	*Pseudomonas aeruginosa* (4-MRGN)	TZP, CTX, MEM, IPM, CIP, SXT
14^a^	*Staphylococcus aureus, Moraxella catarrhalis, Streptococcus pneumoniae, Acinetobacter baumanii*	ermC^b^, ermB^b^	*Staphylococcus aureus, Streptococcus pyogenes*	P, E, CC, TE, SXT, CIP
15	*Streptococcus pneumoniae*	ermB^b^	*Streptococcus anginosus*	CIP
16^a^	*Streptococcus pneumoniae*	ermB^b^	*Citrobacter freundii*	SAM
17	*Moraxella catarrhalis, Serratia marcescens*	none	*Serratia marcescens*	SAM, CXM
18	*Streptococcus pneumoniae, Klebsiella oxytoca*	ermC, mefA^b^, ermB^b^, sul1	*Enterobacter cloacae*	SAM
19	*Klebsiella pneumoniae, Staphylococcus aureus*	ermC^b^, mefA, ermB, dha, ebc	*Enterobacter cloacae*	SAM, CXM
20^a^	*Serratia marcescens, Pseudomonas aeruginosa*	gyrA83_2, gyrA87_2, parC	*Serratia marcescens, Klebsiella oxytoca*	SAM, CXM, SAM, TZP, CXM
21^a^	*Escherichia coli, Haemophilus influenzae, Morganella morganii*	dha	*Proteus mirabilis, Escherichia coli, Morganella morganii*	SXT, SAM, SAM, CXM
22	*Moraxella catarrhalis, Streptococcus pneumoniae, Acinetobacter baumanii, Haemophilus influenzae*	ermB^b^	none	none
23^a^	*Haemophilus influenzae*	mefA, ermB, tem^b^	*Proteus mirabilis*	none
24	*Serratia marcescens, Stenotrophomonas maltophilia, Klebsiella oxytoca*	none	*Serratia marcescens, Raoultella ornithinolytica*	SAM, CXM, MXF, AM, CXM

*AM* Ampicillin, *SAM* Ampicillin-Sulbactam, *CAZ* Ceftazidime, *CTX* Cefotaxime, *CXM* Cefuroxime, *CIP* Ciprofloxacin, *CC* Clindamycin, *SXT* Cotrimoxazole, *E* Erythromycin, *GM* Gentamicin, *IPM* Imipenem, *MEM* Meropenem, *MXF* Moxifloxacin, *OX* Oxacillin, *P* Penicillin G, *TZP* Piperacillin-Tazobactam, *TE* Tetracycline

^a^ Partly test failure mPCR

^b^ Resistance marker encoding for a drug resistance with potential clinical relevance for a pathogen, detected by mPCR

**Fig. 2:**
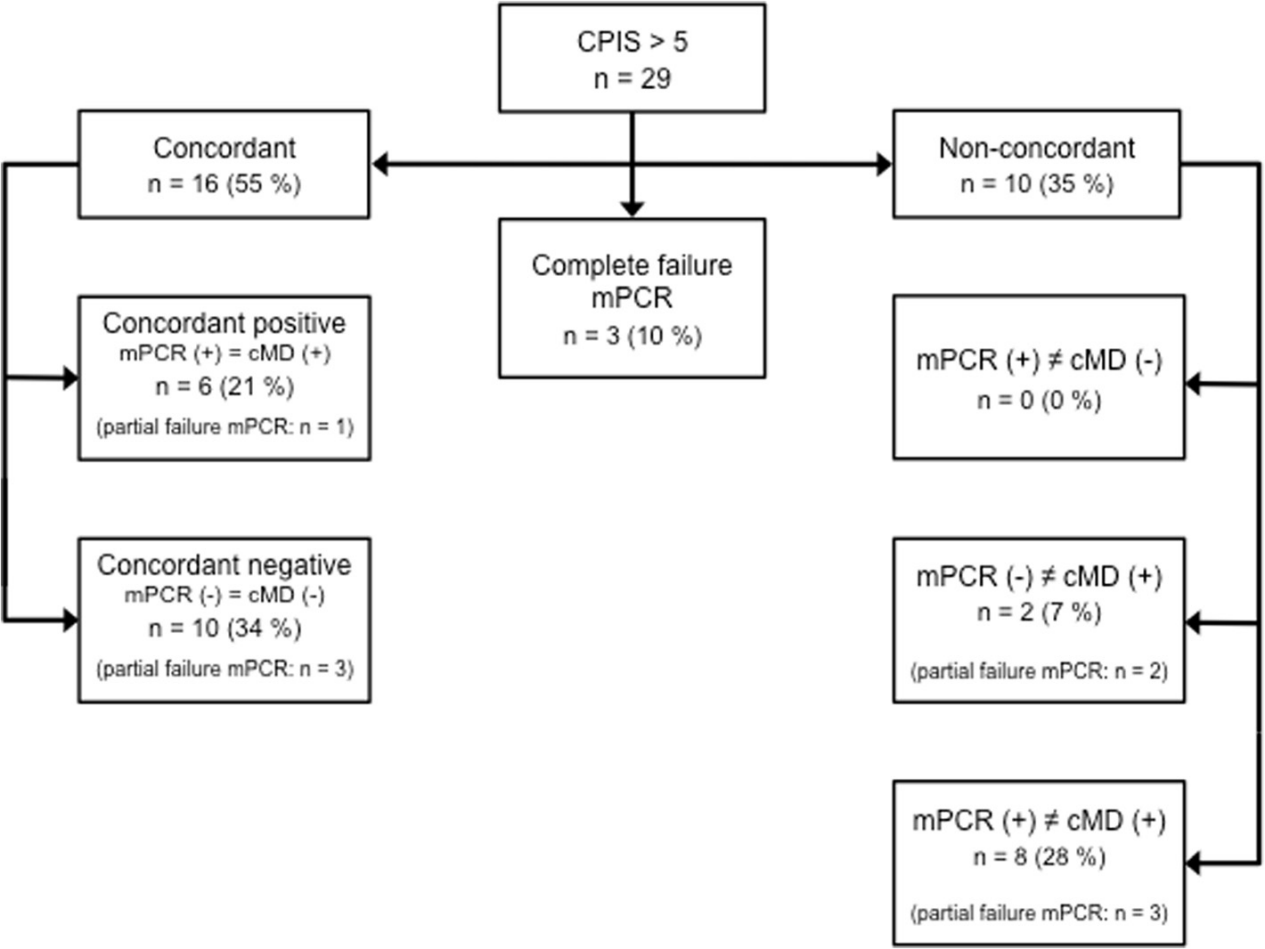
Results in patients with a CPIS > 5

In the sub-group of patients with a CPIS > 5 (*n* = 29) mPCR provided valid results for one or more areas of the test panel in 26 cases. Complete test failure occurred in three patients (10 %). Partial test failure was seen in nine cases (31 %). In 16 patients (55 %) results of mPCR and cMD were concordant. These included the six patients (21 %) in which both tests were concordant positive and ten patients (34 %) were both tests were concordant negative. Non-concordant results occurred in ten patients (35 %), including the two cases where cMD alone detected a pathogen and eight cases (28 %) with a mismatch between mPCR and cMD. Figure 2 summarizes the results for the subgroup with a CPIS > 5.

## Discussion

It was the purpose of this study to evaluate the performance of a new point-of-care multiplex PCR device for rapid microbial testing in the setting of HAP at the ICU. Therefore we performed a pilot study in a real-life ICU environment.

As expected mPCR provided much shorter turnaround times than cMD. The very long mean turnaround time of 71.2 h for cMD reflects the fact that we solely surveyed the time from sampling to the complete cMD results including antibiotic resistance testing. We did not measure turnaround times of incomplete cMD results (such as gram staining results) that are generally available much earlier, usually within a few hrs after sample taking.

With 6.5 h, the mean turnaround time of mPCR was very short. According to the manufacturer one test runs 4.5 h, consisting of 0.5 h for sample lysis and 4 h for amplification. However, the lysated samples needed to be transferred from the lysator into the amplification device. Turnaround times for mPCR in the point-of-care setting therefore depended on (1) how quick after sampling mPCR was started and, after 30 min of lysation, (2) how quick the sample was transferred into the amplification device. Under the real-life circumstances of a busy ICU this explains the variation of turnaround times of the mPCR tests. In a recently published study the median “time to result” for the system has been stated with only 5.2 h [8]. However, for this study Schulte et al. did not operate the system as a point-of-care system at an ICU and therefore mPCR test were started only “on the same day” as cMD was started. Our study, therefore, is the first that provides reliable turnaround times in an ICU point-of-care setting.

We found concordant results (cMD = mPCR) in every second patient. A slightly higher rate of concordant results was found in patients with a high probability for HAP (CPIS > 5). Most non-concordant results (cMD ≠ mPCR) were caused by a mismatch between both methods.

A main reason for non-concordant results might be the inacceptable high number of partially failed mPCR tests. Partial test failure occurred when one or more areas of the test panel failed to produce valid results. Complete test failure occurred when not a single area of the panel produced valid results. One might argue that the complete failed mPCR tests should also be counted as non-concordant results, which makes the performance of the device even more disappointing.

mPCR aims to be a point-of-care test in addition to cMD with the goal to start adequate antibiotic therapy more early in critical ill patients. However, we do not expect this technology to substitute the well-established methods of cMD in recent years. Costs for mPCR testing therefore will be additional diagnostic costs and need to be reasonable for intensive care practitioners. Improvement of the methodology of the system for a better technical performance with less invalid test results would be a first step in that direction. There is a need for larger and interventional trials to show sensibility and specificity of the test in a point-of-care setting and to prove the influence of mPCR diagnostic on the outcome of pneumonia patients.

Methodological differences may also contribute to the high rate of non-concordant results in our study: The exact differentiation of *Streptococcus* species by genomic methods for instance is known to be difficult and some of the many *Streptococcus pneumoniae* mPCR results might reflect this problem [9].

*Haemophilus influenzae* and *S. pneumoniae* furthermore are susceptible for non-optimal conditions as they occur during transport for cMD. Aside from that, mPCR is able to detect pathogens even when only few (not necessarily vital) individuals are present in a specimen [10]. Therefore, the number of identified organisms might differ in both methods. Interpretation of the results should anticipate these and other differences and limitations of both methods.

In this context it is important to notice that mPCR does not provide any information on the presence of residential flora in the investigated specimen. However, the detection of an indicator for residential flora would be useful to evaluate the quality of the specimen, especially when they are taken via nasopharyngeal aspiration in non-intubated patients.

Furthermore some resident organisms do carry relevant resistance markers that can lead to a relevant misjudgment of mPCR results. The resistance marker gene mecA for example encodes resistance to methicillin and is found in both *Staphylococcus aureus* and *Staphylococcus epidermidis*. Unfortunately *S. aureus* alone is part of the mPCR panel. Therefore it is not possible for the user to distinguish whether the detection of mecA in a sample reflects relevant information on the resistance pattern of an etiologic pathogen (e.g. MRSA) or if it implicates specimen contamination by resident organisms such as *S. epidermidis* (false positive MRSA) [11]. Case no. 12 in Table 4 gives an example for such a constellation. Therefore we suggest an extension of the mPCR panel for bacteria representing residential flora (e.g. *S. epidermidis*) to allow the user to distinguish.

In only six cases both methods detected the same bacteria. However, we found three multidrug-resistant bacteria strains in these patients (see cases 1, 4 and 6 in Table 4). Patients suffering from an infection caused by multidrug-resistant microbes have the highest potential to benefit from rapid and reliable pathogen identification. Early administration of the adequate antibiotic is known to have major impact on the prognosis of these patients [12]. Under this assumption, a short diagnostic turnaround time could make a life-saving difference.

Our results confirmed that point-of-care mPCR has the potential to provide microbial testing with significant shorter turnaround times than conventional methods. However, 10 % of the mPCR tests in our study did completely fail. Another 30 % of the mPCR tests partially failed. In other words only 60 % of the mPCR tests were valid and therewith the performance of the device was not acceptable. Schulte et al. recently published a rate of not more than 65.7 % valid results in their study of 739 mPCR tests [8]. Our results do confirm these findings. A reliable technical performance is essential for the successful introduction a new diagnostic tool into everyday clinical use. Besides the medical implications of such an unreliable technical performance, it will be hard for hospitals to legitimate the costs for such a system and for the mPCR tests.

The results of our study were strongly influenced by the poor performance of the system. This fact underlines the pilot character of the study. However, from our point of view even the results of such a small observational study can help to assess the value of a new method for a specialized field of medical practice, such as intensive care medicine.

## Conclusions

The Unyvero mPCR device allowed point-of-care microbial testing with short turnaround times. Therefore it could be a useful addition to cMD because it has the potential to reduce the time to the start of an adequate antibiotic therapy, especially when multidrug-resistant bacteria cause pneumonia.

The actual technical performance of the system is poor and needs to be improved before more research is done on its influence on patient’s outcome. The panel should be extended for at least one indicator for residential flora, which would allow the user to distinguish between pathogens and residential contamination.
